# A Novel Two-Stage Refine Filtering Method for EEG-Based Motor Imagery Classification

**DOI:** 10.3389/fnins.2021.657540

**Published:** 2021-09-01

**Authors:** Yuxin Yan, Haifeng Zhou, Lixin Huang, Xiao Cheng, Shaolong Kuang

**Affiliations:** ^1^The First Affiliated Hospital of Soochow University, Soochow University, Suzhou, China; ^2^College of Mechanical and Electrical Engineering, Soochow University, Suzhou, China; ^3^Applied Technology College of Soochow University, Suzhou, China

**Keywords:** electroencephalogram, motor imagery, 3D representation, multi-branch structure, two-stage refine filtering

## Abstract

Cerebral stroke is a common disease across the world, and it is a promising method to recognize the intention of stroke patients with the help of brain–computer interface (BCI). In the field of motor imagery (MI) classification, appropriate filtering is vital for feature extracting of electroencephalogram (EEG) signals and consequently influences the accuracy of MI classification. In this case, a novel two-stage refine filtering method was proposed, inspired by Gradient-weighted Class Activation Mapping (Grad-CAM), which uses the gradients of any target concept flowing into the final convolutional layer to highlight the important part of training data for predicting the concept. In the first stage, MI classification was carried out and then the frequency band to be filtered was calculated according to the Grad-CAM of the MI classification results. In the second stage, EEG was filtered and classified for a higher classification accuracy. To evaluate the filtering effect, this method was applied to the multi-branch neural network proposed in our previous work. Experiment results revealed that the proposed method reached state-of-the-art classification kappa value levels and acquired at least 3% higher kappa values than other methods This study also proposed some promising application scenarios with this filtering method.

## Introduction

Cerebral stroke (Albers and Olivot, 2007; Menon and Demchuk, 2011) is one of the most common diseases, and disorder in functions related to language and motor makes it hard for stroke patients to live a normal life. It is possible to recognize the intention of stroke patients with the development of brain–computer interface (BCI), which is based on the phenomenon of event-related synchronization (ERS) or event-related desynchronization (ERD) (Neuper et al., 2006; Wilson et al., 2019) in electroencephalogram (EEG) (Tanaka et al., 2005). In this case, the task of motor imagery (MI) classification (Qin et al., 2004; Herman et al., 2008; Taran and Bajaj, 2019; Kato et al., 2020) is carried out and a lot of achievements had been achieved. However, it is still a great challenge to classify the EEG signals accurately. To enhance the accuracy of MI classification and consequently improve the performance of BCI (Schalk et al., 2004; Ge et al., 2019), a large amount of the methods had been proposed by researchers. All the MI classification methods can be generally divided into two categories: Common Spatial Pattern (CSP) (Kang et al., 2009; Lu et al., 2010)-based methods, such as Filter Bank Common Spatial Pattern (FBCSP) (Ang et al., 2012) and Common Spatio-Spectral Pattern (CSSP) (Lemm et al., 2005); and Deep learning based methods (Suwicha et al., 2014; Schirrmeister et al., 2017), such as C2CM (Sakhavi et al., 2018), Compact convolutional neural network (Lawhern et al., 2016), and shallow ConvNet (Schirrmeister et al., 2017).

No matter with which method to carry out MI classification, however, appropriate filtering, which suppresses high-amplitude noise and channel saturation, is also needed (Benigno et al., 2021). In FBCSP (Ang et al., 2012), nine band-pass filters covering the range of 4–40 Hz were used and a spatial filtering using the CSP method followed by each band-pass filter. Various configurations proved to be as effective because these frequency ranges yielded a stable frequency response. Based on FBCSP, C2CM (Sakhavi et al., 2018) is a successful example that combines conventional method and deep learning. This method convolutes time features and spatial features separately, which achieved good performance but increased more parameters. Similarly, in shallow ConvNet (Schirrmeister et al., 2017), FBCSP was also adopted for data processing. A bandpass filtering and the CSP spatial filter are used in the network’s first two layers. The classification results are then computed with the following convolution layers and pooling layers. It is more reasonable than FBCSP because the shallow ConvNet embedded all the computation process in one network and the parameters can be optimized together to acquire a better result. These methods have acquired a high accuracy of MI classification but no further study for the influence of filtering was carried out.

There are also several special filtering methods. In Schrödinger filtering (Benigno et al., 2021), gradient artifact spikes were removed and EEG signals were preserved and templates or references of the artifact or signal were used in this algorithm. Meanwhile, evoked activity was not affected in the filtering process, which proved the robustness of this method. However, this method was based on the semi-classical signal analysis (SCSA), which is young and needs to be studied actively to acquire better performance. Bayesian filtering (Miran et al., 2018) was a different filtering method that decoded real-time auditory attention from EEG and alleviated the need for large training datasets compared with other existing methods. This method is complicated in application to some extent. In clustering-based feature (Yu et al., 2020), the underlying structure of EEG data was explored and the data feature was optimized with a cluster-based multi-task learning algorithm that enhances the accuracy of classification. For the purpose of boosting the classification accuracy, a multi-scale optimization (MSO) of spatial patterns (Zhang et al., 2019; Jiao et al., 2020) was proposed, which optimizes filter bands via multi-view learning within CSP. This method also acquired good results in MI-related EEG datasets with the filtering method.

All the methods mentioned above tried to filter the EEG signals before MI classification, and these methods usually divided the filtering process into several steps, which seems to be complicated. In this study, a novel two-stage refine filtering method was proposed, inspired by the discussion of Gradient-weighted Class Activation Mapping (Grad-CAM) (Selvaraju et al., 2020) in our previous work (Xinqiao et al., 2019; Zhou et al., 2019), which proposed a 3D representation of EEG and constructed a multi-branch convolutional neural network for MI classification. Gradient-weighted Class Activation Mapping makes a good visual explanation by highlighting the important regions of predicted images according to the last convolutional layer of the network. In this case, we considered whether it is possible to improve the performance of our network by preserving the useful frequency (which means these frequencies contribute a lot to correct classification results) and suppress useless frequency (which means these frequencies contribute nothing to correct results).

The two-stage refine filtering method can be divided into two stages: In the first stage, raw EEG data were trained in the network and the frequency bands to be filtered were selected according to the Grad-CAM results of the last convolutional layers. In the second stage, the EEG data were filtered with the selected frequencies and put into the network again. This method was applied to the dataset of BCI competition IV-2a (Ang et al., 2012). The MI classification results of two stages were recorded and the result of the second stage was better than that of the first stage.

The remainder of this article is organized as follows. *Methods* gives a description of the MI classification strategy. The experiment and results are presented in *Experiment and Results*. The discussion is shown in *Discussion*. *Conclusion* concludes the article.

## Materials and Methods

In this section, detailed configurations of the filtering method including the two-stage filtering process and the frequency selecting principles in this process are illustrated.

### Two-Stage Refine Filtering Process

**FIGURE 1:**
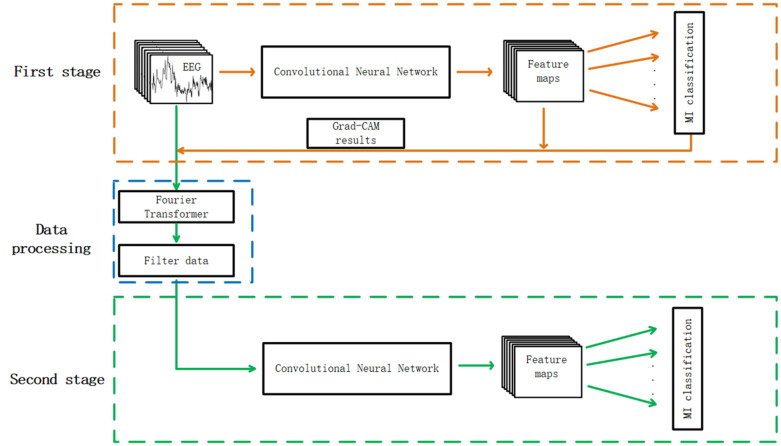
The whole architecture of the two stage refine filtering method. The convolutional neural network of the first stage was the same as the one of the second stage.

The whole process of this method is shown in Figure 1. The classification system was divided into two stages. In the first stage, the EEG data without filtering were fed into a convolutional neural network to complete the first training and the weights were saved. The Grad-CAM results were acquired according to the feature maps and the MI classification results. Then, it can be figured out whether training data contribute a positive or negative weight to correct classification results. In the data processing process, the frequencies to be filtered were determined according to the Grad-CAM results and detailed frequency selecting principles are illustrated in the next section. In the second stage, the raw data were filtered with the frequencies selected according to the results of first stage. The filtered data were used as input in the second training process.

### Frequency Selecting Principles

**FIGURE 2:**
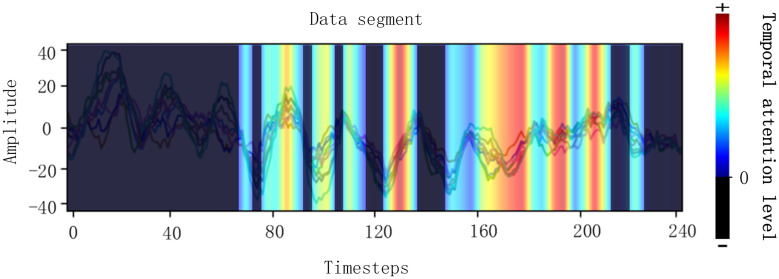
Visualizations of Grad-CAM for EEG signals. The bright regions mean EEG in this region contributes to correct classification results. The dark regions mean EEG in this region contributes a negative weight to correct classification results.

After the first training process with the backbone network, the Grad-CAM, which is shown in Figure 2, is carried out to judge which part of a cropped data segment is beneficial to the correct results. The Grad-CAM was determined according to the classification results of the first stage and Figure 2 shows that the signals with a bright color contribute to correct results and the signals with a dark color contribute nothing to correct results. The signals with a bright color were called useful data, which contribute a positive weight to correct classification results, and the dark one was called useless data, which contribute negative weights to correct results. Then, the data were transformed with Fourier transformation to extract frequency information. The frequency of useful data that contribute to correct results is named good frequency, and the frequency of useless data that contribute nothing to correct results is named bad frequency. It is obvious that different data samples have different random “useful” or “useless” time intervals and there are many intervals in each segment to be calculated. Considering the huge amount of training data, a tenth of the data were sampled for Grad-CAM.

#### Details of the Selecting Process

**FIGURE 3:**
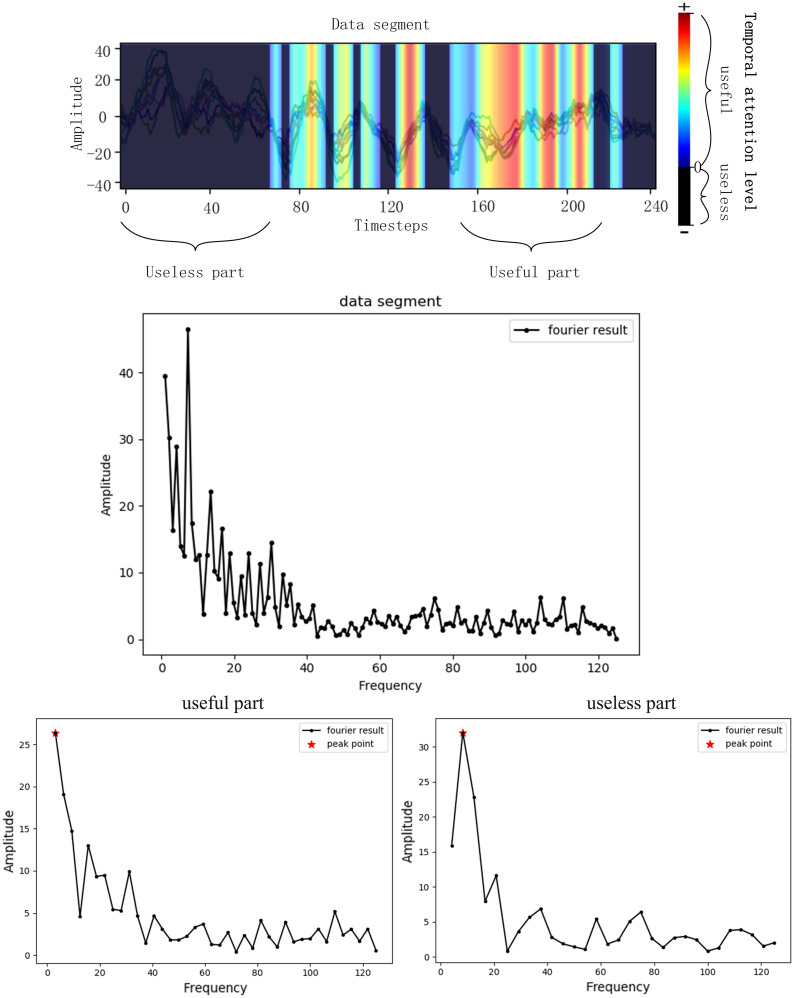
Frequency selecting process. The Fourier transformer results of a data segment, a useless part of the segment and a useful part of the segment was presented. The frequency of peak point was the selected useful frequency or useless frequency.

As is introduced before, the EEG signals are assessed with Grad-CAM and then the bad and good frequencies of the signals are recorded, respectively, which is illustrated in Figure 3. A useless part of the data segment was Fourier transformed and the frequency with highest amplitude was recorded as the useless frequency considering that the frequency with the highest amplitude may be most representative. The useful part was processed the same as the useless part. The bad frequency data cannot just be filtered because many frequencies belong to both good and bad frequency due to the huge amount of EEG and the inaccuracy of the data acquisition process. The low frequencies will always be chosen due to the 1/f characteristic of the power spectrum, and in this case, many low frequencies repeated in both good and bad frequencies. To get a better performance of the network by filtering bad frequency, the bad frequency needs to be selected further according to its amount.

**FIGURE 4:**
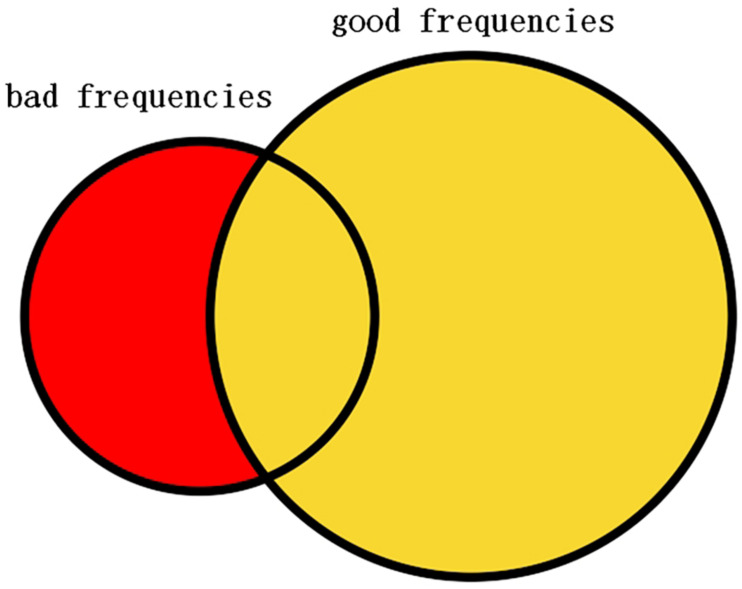
Left circle represents the selected bad frequencies. Right circle represents the selected good frequencies. Red part of the left circle represents the selected frequencies to be filtered.

In this study, the selected frequencies were collected and the frequencies that belong to bad frequencies but do not belong to good frequencies are selected as the filtering frequency that is illustrated in Figure 4. The bad and good frequencies are sorted, respectively, in descending order according to their amount. To avoid filtering out useful signals, a small amount of the bad frequencies ranking ahead were selected and a large amount of the good frequencies ranking ahead were selected. Having tried several configurations, in this study, the top 20 bad frequencies are selected and the top 100 good frequencies are selected. Among the 20 bad frequencies, frequencies that do not belong to good frequencies are selected as the filtering frequency.

## Experiment and Results

### Experiment Setup

The methods proposed above are evaluated on BCI competition IV dataset 2a (Ang et al., 2012). The EEG dataset was recorded with 22 Ag/AgCl electrodes that are distributed according to the international 10–20 system. The data acquisition experiment prompts nine subjects to perform four different tasks named imagery movement of left hand, right hand, both feet, and tongue. For each subject, two sessions on different days were recorded and each session consists of 288 trials. Each trial belongs to one of the four classes and each trial consists of a fixation process of 2 s, a cue time of 1.25 s, and a MI time of 4 s; a short break followed after the MI process. A band-pass filter between 0.5 and 100 Hz was applied to the signals, and a notch filter of 50 Hz was taken to suppress line noise.

In this study, the 1.25-s period of EEG data after the visual cue was taken as experiment data. The EEG was then represented with the 3D representation method mentioned above and the label corresponding to the cropped EEG was presented with a one-hot-vector format.

To evaluate the experiment results, the 10-fold cross-validation method was used. All training data and testing data of BCI IV dataset 2a are combined and then divided into 10 subsets randomly. In each run, nine subsets were used to train and the other one was used as validation data. The final results were obtained by averaging 10 validation results. After the date was filtered, the filtered data were also evaluated with the above method. The *p*-values presented in the experiment were calculated from a two-tailed paired *t*-test.

### Backbone Network

**FIGURE 5:**
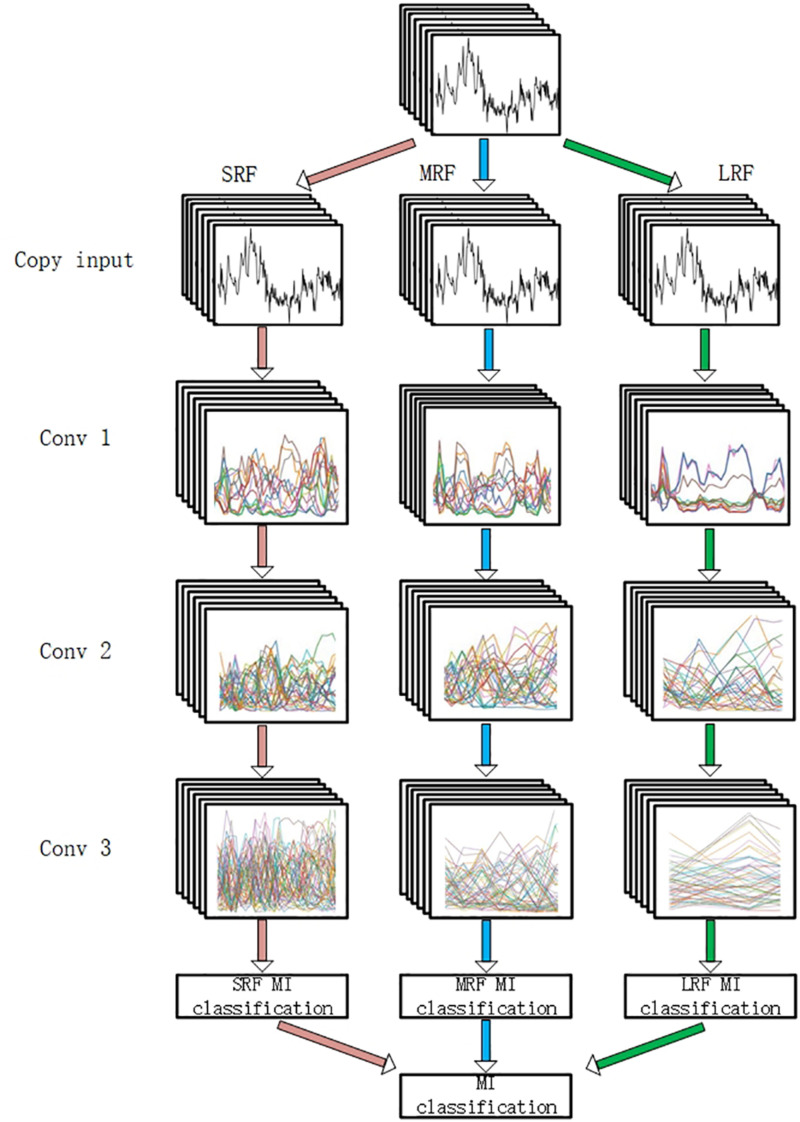
The backbone network. “SRF,” “MRF,” and “LRF” means three different branch networks with different receptive field. The input was copped as “copy input.” “Conv1,” “Conv2,” and “Conv3” mean three convolutional process. “SRF MI classification,” “MRF MI classification,” and “LRF MI classification” represents three classification results of three branches. The MI classification was computed according to three results.

To evaluate the filtering method, a MI classification structure is needed. In this study, a multi-branch convolutional neural network proposed in our former work (Xinqiao et al., 2019) was adopted. As is shown in Figure 5, our backbone network was made up of three CNNs with different receptive fields and the branches are, respectively, named small receptive field network (SRF), medium receptive field network (MRF), and large receptive field network (LRF). The input of the network was a concatenation of 22 electrode signals that contain both temporal and spatial information.

Three branches were fed, respectively, with 3D EEG proposed in our previous work and small convolutional filters are adopted in the light of VGG’s architecture. Forward and back propagation is carried in different branches simultaneously and a soft-max (Liu and Liu, 2017) activation is set in the end of each branch. The final classification result is acquired by summing the branch networks’ respective results and putting the summing result into an additional soft-max activation.

#### Modification of Previous Network

In our previous study, it was discussed that different branches focus on different temporal information. For the purpose of recording each Grad-CAM result of all electrodes’ signals in each branch and filtering them in each branch, the input needs to be copied into three copies. In this circumstance, the first shared convolution layer of the previous network was replaced with three single convolution layers. To reduce the computation cost enhanced by this modification, the number of the dense layer’s nodes was reduced. The convolution layer’s parameters are presented in Table 1.

**TABLE 1:** Three convolution layers’ parameters of three branches.

Conv layer	SRF	MRF	MRF
Conv1	Size: 3 × 3 × 5	Size: 3 × 3 × 5	Size: 3 × 3 × 5
	Strides: 2, 2, 4	Strides: 2, 2, 4	Strides: 2, 2, 4
	Filters: 16	Filters: 16	Filters: 16
Conv2	Size: 2 × 2 × 1	Size: 2 × 2 × 3	Size: 2 × 2 × 5
	Strides: 2, 2, 1	Strides: 2, 2, 2	Strides: 2, 2, 4
	Filters: 32	Filters: 32	Filters: 32
Conv3	Size: 2 × 2 × 1	Size: 2 × 2 × 3	Size: 2 × 2 × 5
	Strides: 2, 2, 1	Strides: 2, 2, 2	Strides: 2, 2, 4
	Filters: 64	Filters: 64	Filters: 64

*“Size” means the height, width, and depth of the 3D convolution window. “Strides” means the strides of the convolution window along each dimension. “Filters” means the number of output filters in each convolution layer.*

### Two-Stage Refine Training Evaluation

#### MI Classification Experiments

The 10-fold validation results of the multi-branch network in the first stage and the second stage are presented in Table 2. In this table, MB-I represents the first classification stage and MB-II represents the second classification stage. MB-I and MB-II are two stages of one system whose former stage was fed with raw training data and the latter stage was fed with filtered training data. The results of the two stages were compared to determine whether the method can improve the classification results.

**TABLE 2:** Comparison of 10-fold cross-validation results training with raw data and filtered data.

Subject	MB-I	MB-II
1	74.8125	75.3158
2	58.923	60.429
3	78.297	79.221
4	69.901	70.601
5	67.083	67.989
6	67.699	68.461
7	74.142	75.382
8	76.705	77.047
9	82.859	83.461
Mean	72.269	73.101
Standard deviation	7.156	6.937
*p*-value	1.3E-04	–

*p-value means the significant difference of the cross-validation results carried by the same network training with raw data and filtered data.*

Comparing the results of nine subjects between MB-I and MB-II, it is obvious that all subjects with MB-II perform better than with MB-I on testing accuracy and the mean value of MB-II is nearly 1% higher than MB-I with *p*-value < 0.01. The standard deviation value of MB-I is 0.2% higher than MB-II, which means the filtered data are more stable than raw data in classification tasks.

#### Learning Process Visualization

**FIGURE 6:**
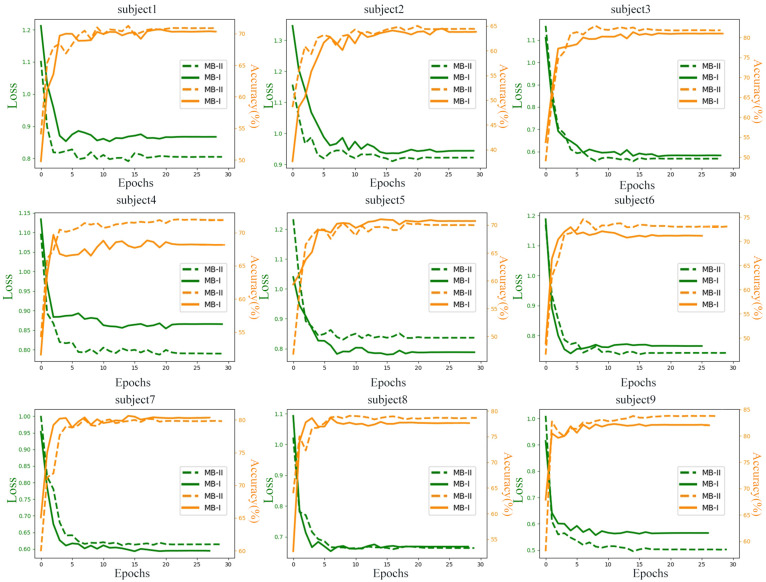
Test loss and accuracy of nine subjects in thirty epochs.

To explore the difference of learning process between raw data and filtered data, the data were trained for 30 epochs and the testing losses (the Negative log-likelihood cost) and accuracies of all subjects are monitored and recorded in Figure 6. The testing data were random one-fold data of each subject. MB-I represents the first classification stage and MB-II represents the second classification stage. The green line represents the loss in training process and the orange line represents the accuracy in training process. The left axis of each subplot represents the loss value of each subject and the right axis represents the accuracy value.

It is obvious that the accuracy of MB-II is higher than MB-I except for most subjects. For subject 4, the performances of the network evaluated with filtered data are outstanding with an accuracy of 4% higher than with raw data. Actually, the results of subject 4 ranges widely in the 10-fold validation process, which means that the MB-II did not always perform much better in 10-fold validation results; however, the mean accuracy of MB-II was higher than MB-I as is illustrated in Table 2.

For subject 1, subject 6, and subject 8, the MB-II acquired a lower testing accuracy and a higher testing loss than MB-I in early epochs. However, in the end of the training process, the MB-II performed better than MB-I, which means the filtering method is useful for enhancing classification accuracy.

For subject 2 and subject 3, the accuracy of MB-I and MB-II is nearly the same but the network evaluated with filtered data acquired lower testing loss, which may benefit from filtering several bad frequencies.

### Comparison With the State-of-the-Art

Three state-of-the-art MI classification methods and our formal classification method are compared with the method proposed in this study. We firstly give a brief description of other methods having been introduced in the *Introduction* section and then analyze the kappa value (Lai et al., 2016; Wang et al., 2019) of different methods, which is defined to evaluate classification accuracy. Results of all methods are recorded in Table 3. “MB-M” represents the modified multi-branch network used in this study and “MB” represents the multi-branch network in our previous study.

**TABLE 3:** Comparison of the kappa value with the state-of-the-art methods.

Subject	MB-M	MB	FBCSP	Shallow ConvNet	C2CM
1	0.698	0.699	0.68	0.820	0.833
2	0.523	0.459	0.42	0.432	0.537
3	0.787	0.788	0.75	0.835	0.87
4	0.629	0.594	0.48	0.621	0.556
5	0.669	0.647	0.40	0.490	0.5
6	0.622	0.538	0.27	0.380	0.273
7	0.642	0.653	0.77	0.898	0.861
8	0.746	0.702	0.76	0.758	0.778
9	0.825	0.713	0.61	0.657	0.727
Mean	0.682	0.644	0.571	0.655	0.659
Standard deviation	0.093	0.100	0.184	0.188	0.204

Filter Bank Common Spatial Pattern: FBCSP was proposed in Ang et al. (2012) and is capable of autonomously selecting the proper subject-specific frequency range for bandpass filtering. The method performed best on the BCI Competition IV 2a dataset in competition.

Shallow ConvNet (Schirrmeister et al., 2017): Inspired by FBCSP, the network adopts bandpass and CSP spatial filters in the first two layers so as to optimize all parameters jointly.

C2CM (Sakhavi et al., 2018): C2CM adopts the strategy of breaking up 2D convolutions into two 1D convolutions and filtering data with FBCSP. This method makes the network more flexible for separating the information of time and space but increases the computation parameters for an additional layer.

As is shown in Table 3, the kappa value of MB-M is higher than MB for most subjects. For subject 6 and subject 9, the kappa value of MB-M is nearly 1% higher than MB, which reveals the advantage of the two-stage refine training method. The MB-M also outperforms other state-of-the-art methods with a higher mean value and a lower standard deviation value. For subject 9, the kappa value of MB-M was much higher than other methods, which means several vital bad frequencies were filtered in the second stage.

## Discussion

The proposed two-stage refine filtering method, which is inspired by Grad-CAM, was novel and proved to improve the performance of the multi-branch network proposed in our previous study. In the field of MI classification, correct filtering suppresses the influence of noise and makes the feature extracting process easier. Consequently, the MI classification results are improved. According to the experiment results, it can be inferred that the filtered training data are more stable than the raw training data and the method is robust in different subjects.

It is special for this method to improve the performance of a network according to its mechanism. In other words, the method improves the classification results according to the characteristics of training data with little artificial intervention. In this case, the filtering method could be commonly used in the majority of deep learning-related tasks. To further evaluate the method, the filtering method can be applied to different EEG-related networks proposed by others. Moreover, the method should not only be effective on MI classification since the filtering range is selected according to the performance of the network. Similarly, applying the method to other fields such as voice processing is an advisable attempt.

It is also worth mentioning that this method is actually an iterable process, which means we can filter the data after the last training process according to the Grad-CAM of the last classification results. However, multiple iterations may lead to overfitting in the training process and thus influence the correct MI classification. In this case, an appropriate amount of frequencies to be filtered is needed and higher accuracy of MI classification may be acquired in this way.

## Conclusion

In this study, a two-stage refine filtering method was proposed for MI classification inspired by Grad-CAM. The method was applied to the multi-branch network and proved to improve the performance of the network. This method is considered as a universal method and promising in many other fields.

## Data Availability Statement

The original contributions presented in the study are included in the article/Supplementary Material, further inquiries can be directed to the corresponding author/s.

## Author Contributions

All authors listed have made a substantial, direct and intellectual contribution to the work, and approved it for publication.

## Conflict of Interest

The authors declare that the research was conducted in the absence of any commercial or financial relationships that could be construed as a potential conflict of interest.

## Publisher’s Note

All claims expressed in this article are solely those of the authors and do not necessarily represent those of their affiliated organizations, or those of the publisher, the editors and the reviewers. Any product that may be evaluated in this article, or claim that may be made by its manufacturer, is not guaranteed or endorsed by the publisher.
