# Supplementary material for: A Novel Two-Stage Refine Filtering Method for EEG-Based Motor Imagery Classification
Source: Front Neurosci. 2021 Sep 1;15:657540. doi: 10.3389/fnins.2021.657540 (PMC8440963; doi:10.3389/fnins.2021.657540)
Supplement: Supplementary file 1 [file Data_Sheet_1.PDF]

BCI Competition 2008 – Graz data set A

C. Brunner[1], R. Leeb[1], G. R. Müller-Putz[1], A. Schlögl[2], and G. Pfurtscheller[1]

[1]Institute for Knowledge Discovery, Graz University of Technology, Austria
[2]Institute for Human-Computer Interfaces, Graz University of Technology, Austria

Experimental paradigm

This data set consists of EEG data from 9 subjects. The cue-based BCI paradigm consisted of four different motor imagery tasks, namely the imagination of movement of the left hand (class 1), right hand (class 2), both feet (class 3), and tongue (class 4). Two sessions on different days were recorded for each subject. Each session is comprised of 6 runs separated by short breaks. One run consists of 48 trials (12 for each of the four possible classes), yielding a total of 288 trials per session.

At the beginning of each session, a recording of approximately 5 minutes was performed to estimate the EOG influence. The recording was divided into 3 blocks: (1) two minutes with eyes open (looking at a fixation cross on the screen), (2) one minute with eyes closed, and (3) one minute with eye movements. The timing scheme of one session is illustrated in Figure 1. Note that due to technical problems the EOG block is shorter for subject `A04T` and contains only the eye movement condition (see Table 1 for a list of all subjects).

The subjects were sitting in a comfortable armchair in front of a computer screen. At the beginning of a trial ($t = 0\,\mathrm{s}$), a fixation cross appeared on the black screen. In addition, a short acoustic warning tone was presented. After two seconds ($t = 2\,\mathrm{s}$), a cue in the form of an arrow pointing either to the left, right, down or up (corresponding to one of the four classes

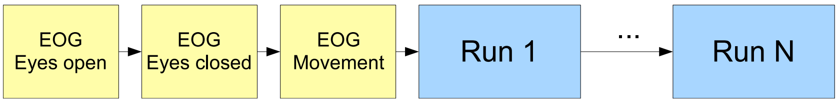

Figure 1: Timing scheme of one session.

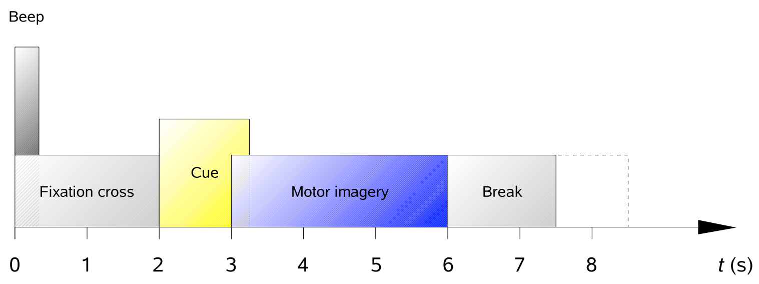

Figure 2: Timing scheme of the paradigm.

left hand, right hand, foot or tongue) appeared and stayed on the screen for 1.25 s. This prompted the subjects to perform the desired motor imagery task. No feedback was provided. The subjects were ask to carry out the motor imagery task until the fixation cross disappeared from the screen at $t = 6$ s. A short break followed where the screen was black again. The paradigm is illustrated in Figure 2.

Data recording

Twenty-two Ag/AgCl electrodes (with inter-electrode distances of 3.5 cm) were used to record the EEG; the montage is shown in Figure 3 left. All signals were recorded monopolarly with the left mastoid serving as reference and the right mastoid as ground. The signals were sampled with 250 Hz and bandpass-filtered between 0.5 Hz and 100 Hz. The sensitivity of the amplifier was set to 100 µV. An additional 50 Hz notch filter was enabled to suppress line noise.

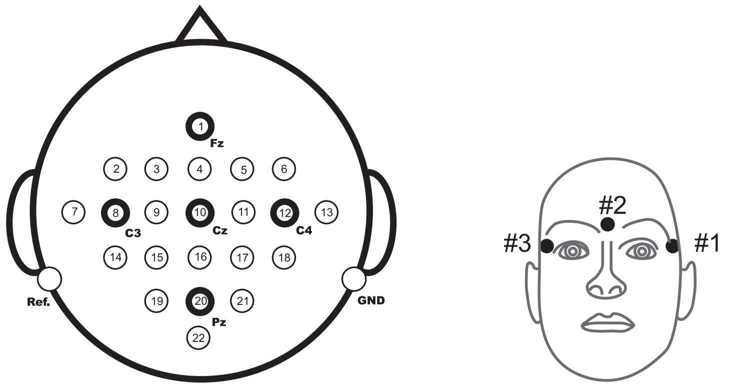

Figure 3: Left: Electrode montage corresponding to the international 10-20 system. Right: Electrode montage of the three monopolar EOG channels.

In addition to the 22 EEG channels, 3 monopolar EOG channels were

recorded and also sampled with 250 Hz (see Figure 3 right). They were bandpass filtered between 0.5 Hz and 100 Hz (with the 50 Hz notch filter enabled), and the sensitivity of the amplifier was set to 1 mV. The EOG channels are provided for the subsequent application of artifact processing methods [1] and must not be used for classification.

A visual inspection of all data sets was carried out by an expert and trials containing artifacts were marked. Eight out of the total of nine data sets were analyzed in [2, 3].

Data file description

All data sets are stored in the General Data Format for biomedical signals (GDF), one file per subject and session. However, only one session contains the class labels for all trials, whereas the other session will be used to test the classifier and hence to evaluate the performance. All files are listed in Table 1. Note that the evaluation sets will be made available after the deadline of the competition (except for one file from subject `A01` which serves as an example). The GDF files can be loaded using the open-source toolbox BioSig, available for free at http://biosig.sourceforge.net/. There are versions for Octave[1]/FreeMat[2]/MATLAB[3] as well as a library for C/C++.

ID	Training	Evaluation
1	A01T.gdf	A01E.gdf
2	A02T.gdf	~~A02E.gdf~~
3	A03T.gdf	~~A03E.gdf~~
4	A04T.gdf	~~A04E.gdf~~
5	A05T.gdf	~~A05E.gdf~~
6	A06T.gdf	~~A06E.gdf~~
7	A07T.gdf	~~A07E.gdf~~
8	A08T.gdf	~~A08E.gdf~~
9	A09T.gdf	~~A09E.gdf~~

Table 1: List of all files contained in the data set, the striked out evaluation data sets will be provided after the deadline of the competition. Note that due to technical problems the EOG block is shorter for subject `A04T` and contains only the eye movement condition.

A GDF file can be loaded with the BioSig toolbox with the following command in Octave/FreeMat/MATLAB (for C/C++, the corresponding function `HDRTYPE* sopen` and `size_t sread` must be called):

```
[s, h] = sload('A01T.gdf');
```

[1] http://www.gnu.org/software/octave/
[2] http://freemat.sourceforge.net/
[3] The MathWorks, Inc., Natick, MA, USA

Event type		Description
276	0x0114	Idling EEG (eyes open)
277	0x0115	Idling EEG (eyes closed)
768	0x0300	Start of a trial
769	0x0301	Cue onset left (class 1)
770	0x0302	Cue onset right (class 2)
771	0x0303	Cue onset foot (class 3)
772	0x0304	Cue onset tongue (class 4)
783	0x030F	Cue unknown
1023	0x03FF	Rejected trial
1072	0x0430	Eye movements
32766	0x7FFE	Start of a new run

Table 2: List of event types (the first column contains decimal values and the second hexadecimal values).

Note that the runs are separated by 100 missing values, which are encoded as not-a-numbers (NaN) by default. Alternatively, this behavior can be turned off and the missing values will be encoded as the negative maximum values as stored in the file with:

```
[s, h] = sload('A01T.gdf', 0, 'OVERFLOWDETECTION:OFF');
```

The workspace will then contain two variables, namely the signals `s` and a header structure `h`. The signal variable contains 25 channels (the first 22 are EEG and the last 3 are EOG signals). The header structure contains event information that describes the structure of the data over time. The following fields provide important information for the evaluation of this data set:

```
h.EVENT.TYP
h.EVENT.POS
h.EVENT.DUR
```

The position of an event in samples is contained in `h.EVENT.POS`. The corresponding type can be found in `h.EVENT.TYP`, and the duration of that particular event is stored in `h.EVENT.DUR`. The types used in this data set are described in Table 2 (hexadecimal values, decimal notation in parentheses). Note that the class labels (i. e., 1, 2, 3, 4 corresponding to event types 769, 770, 771, 772) are only provided for the training data and not for the testing data.

The trials containing artifacts as scored by experts are marked as events with the type 1023. In addition, `h.ArtifactSelection` contains a list of all trials, with `0` corresponding to a clean trial and `1` corresponding to a trial containing an artifact.

In order to view the GDF files, the viewing and scoring application SigViewer v0.2 or higher (part of BioSig) can be used.

Evaluation

Participants should provide a continuous classification output for each sample in the form of class labels (1, 2, 3, 4), including labeled trials and trials marked as artifact. A confusion matrix will then be built from all artifact-free trials for each time point. From these confusion matrices, the time course of the accuracy as well as the kappa coefficient will be obtained [5]. The algorithm used for this evaluation will be provided in BioSig. The winner is the algorithm with the largest kappa value `X.KAP00`.

Due to the fact that the evaluation data sets will not be distributed until the end of the competition, the submissions must be programs that accept EEG data (the structure of this data must be the same as used in all training sets[4]) as input and produce the aforementioned class label vector.

Since three EOG channels are provided, it is required to remove EOG artifacts before the subsequent data processing using artifact removal techniques such as highpass filtering or linear regression [4]. In order to enable the application of other correction methods, we have opted for a maximum transparency approach and provided the EOG channels; at the same time we request that artifacts do not influence the classification result.

All algorithms must be causal, meaning that the classification output at time k may only depend on the current and past samples $x_k, x_{k-1}, \ldots, x_0$. In order to check whether the causality criterion and the artifact processing requirements are fulfilled, all submissions must be open source, including all additional libraries, compilers, programming languages, and so on (for example, Octave/FreeMat, C++, Python, ...). Note that submissions can also be written in the closed-source development environment MATLAB as long as the code is executable in Octave. Similarily, C++ programs can be written and compiled with a Microsoft or Intel compiler, but the code must also compile with g++.

References

[1] M. Fatourechi, A. Bashashati, R. K. Ward, G. E. Birch. EMG and EOG artifacts in brain computer interface systems: a survey. Clinical Neurophysiology 118, 480–494, 2007.

[4] One evaluation data set is distributed from the beginning of the competition to enable participants to test their program and to ensure that it produces the desired output.

[2] M. Naeem, C. Brunner, R. Leeb, B. Graimann, G. Pfurtscheller. Seperability of four-class motor imagery data using independent components analysis. Journal of Neural Engineering 3, 208–216, 2006.

[3] C. Brunner, M. Naeem, R. Leeb, B. Graimann, G. Pfurtscheller. Spatial filtering and selection of optimized components in four class motor imagery data using independent components analysis. Pattern Recognition Letters 28, 957–964, 2007.

[4] A. Schlögl, C. Keinrath, D. Zimmermann, R. Scherer, R. Leeb, G. Pfurtscheller. A fully automated correction method of EOG artifacts in EEG recordings. Clinical Neurophysiology 118, 98–104, 2007.

[5] A. Schlögl, J. Kronegg, J. E. Huggins, S. G. Mason. Evaluation criteria in BCI research. In: G. Dornhege, J. del R. Millán, T. Hinterberger, D. J. McFarland, K.-R. Müller (Eds.). Toward brain-computer interfacing, MIT Press, 327–342, 2007.
